# CHA_2_DS_2_-VASc score and left atrial volume dilatation synergistically predict incident atrial fibrillation in hypertension: an observational study from the Campania Salute Network registry

**DOI:** 10.1038/s41598-019-44214-2

**Published:** 2019-05-27

**Authors:** Antonio Rapacciuolo, Costantino Mancusi, Grazia Canciello, Raffaele Izzo, Teresa Strisciuglio, Nicola de Luca, Giuseppe Ammirati, Giovanni de Simone, Bruno Trimarco, Maria-Angela Losi

**Affiliations:** 1 Hypertension Research Center, University of Naples, Federico II, Naples, Italy; 2 Department of Advanced Biomedical Sciences, University of Naples, Federico II, Naples, Italy

**Keywords:** Epidemiology, Atrial fibrillation

## Abstract

Arterial hypertension is a leading risk factor for developing atrial fibrillation. CHA_2_DS_2_-VASc score can help to decide if patients with atrial fibrillation need anticoagulation. Whether CHA_2_DS_2_-VASc may predicts incident atrial fibrillation and how it interacts with left atrial dilatation is unknown. We tested this hypothesis in a large registry of treated hypertensive patients. From 12154 hypertensive patients we excluded those with prevalent atrial fibrillation (n 51), without follow-up (n 3496), or carotid ultrasound (n 1891), and low ejection fraction (i.e. <50%, n 119). A CHA_2_DS_2_-VASc score ≥3 was compared with CHA_2_DS_2_-VASc score ≤2. Incident symptomatic or occasionally detected atrial fibrillation was the end-point of the present analysis. At baseline, 956 (15%) patients exhibited high CHA_2_DS_2_-VASc; they were older, most likely to be women, obese and diabetic, with lower glomerular filtration rate, and higher prevalence of left ventricular hypertrophy, left-atrial dilatation and carotid plaque (all p < 0.005). Prevalent Stroke/TIA was found only in the subgroup with high CHA_2_DS_2_-VASc. During follow-up (median = 54 months) atrial fibrillation was identified in 121 patients, 2.57-fold more often in patients with high CHA_2_DS_2_-VASc (95% Cl 1.71–4.86 p < 0.0001). In multivariable Cox analysis, CHA_2_DS_2_-VASc increased incidence of atrial fibrillation by 3-fold, independently of significant effect of left-atrial dilatation (both p < 0.0001) and other markers of organ damage. Incident AF is more than doubled in hypertensive patients with CHA_2_DS_2_-VASc ≥3. Coexisting CHA_2_DS_2_-VASc score >3 and LA dilatation identify high risk subjects potentially needing more aggressive management to prevent AF and associated cerebrovascular ischemic events.

## Introduction

Arterial hypertension (Hpt) is a major cardiovascular disorder and atrial fibrillation (AF) the most common arrhythmia. These conditions frequently overlaps and their prevalence increases with age. Different risk factors and clinical conditions predispose to the development of AF, but because of its high prevalence, Hpt is still the most common population-attributable risk for the development of this arrhythmia^1^.

Since the prevalence of both medical conditions is increasing paralleling human life expectation, AF in hypertensive patients will become a leading risk factor for cardiovascular morbidity and mortality in the next future^1^.

More than 30% of patient with AF presented with no obvious symptoms or impaired quality of life. Therefore, the first clinical evidence of this ‘silent’ AF could be a thromboembolic event^2^. At least 30% of patients presenting with strokes present previously unrecognized AF^3^. MRI studies revealed that up to 40% of patients with AF had one or more silent cerebral infarcts^4^. The attempt to accurately predict risk of thromboembolic events, and especially of cardioembolic stroke, has been a very important field of scientific interest in the recent years. CHA_2_DS_2_-VASc score has been demonstrated to be useful in early risk stratification of patients with non-valvular AF in terms of intracardiac thrombogenesis and cardioembolism^5^, and allows to start antithrombotic treatments with a net favourable balance between stroke prevention and bleeding complications^6–8^.

Furthermore, in patients with nonvalvular AF and stroke, CHA_2_DS_2_-VASc score was found to be associated with intracerebral atherosclerosis^9^, suggesting that these scores might reflect the global burden of atherosclerosis^10^. This association also suggests that the CHA_2_DS_2_-VASc might be used to stratify CV risk beyond the limits of current guidelines.

In the contest of the Campania Salute Network (CSN) registry, the development of AF has been recently considered as an incident CV event and used as soft end-point. Therefore, given the above rationale, in the present study we assess whether a high CHA_2_DS_2_-VASc score can predict incident AF in a registry of treated hypertensive patients.

## Methods

### Participants

The CSN is an open registry collecting information from general practitioners and community hospitals in the 5 districts of the Campania Region, in Southern Italy. General practitioners and community hospitals are networked with the Hypertension Research Center of the Federico II University Hospital in Naples^4^. The data-base generation of CSN was approved by “Federico II” institutional Ethic Committee and signed informed consent was obtained from all participants. All research was performed in accordance with relevant guidelines/regulations. All hypertensive patients of the network were referred for baseline echocardiograms and carotid ultrasound to our Hypertension Center. Detailed characteristics of CSN population have been previously repeatedly reported^2,11,12^.

From a population of 12154 hypertensive patients we excluded patients with AF at baseline (n 51), no follow-up (n 3496), unavailable carotid ultrasound (n 1891), low left ventricular (LV) ejection fraction, (<50%, n 119). Thus, the study population comprised 6597 hypertensive patients.

### Outcome

All prevalent and incident CV or cerebrovascular events (including AF), were adjudicated by the Committee for Event Adjudication in the Hypertension Research Center. Adjudication was based on patients’ history, contact with the reference general practitioner or community hospital and clinical records documenting the occurrence of the event/arrhythmia^2,13^.

Incident AF was the end-point of the present analysis. Incident AF was demonstrated in symptomatic patients going to the emergency department or in asymptomatic patients during a routine visit. AF was confirmed by an ECG performed at our outpatient clinics or in other hospitals at the time of hospitalization or by the GP at the time of the control visit^2^.

### Measurements and definitions

Diabetes was defined according to 2007 ADA criteria (fasting plasma glucose >125 mg/dl or anti-diabetic treatment)^14^. Obesity was defined as a BMI ≥30 kg/m^2^. Systolic and diastolic BP were measured by standard aneroid sphygmomanometer after 5 minutes resting in the sitting position, according to current guidelines^15^. Glomerular filtration rate (GFR) was measured by the CKD-EPI (Chronic Kidney Disease Epidemiology collaboration) equation^16,17^.

### Echocardiography

Echocardiograms were recorded in our Hypertension Research Center on videotapes, using commercial machines and a standardized protocol, were digitally mastered and read off line by one expert reader under the supervision of a senior faculty member, using dedicated work-stations (MediMatic, Genova, Italy^1^).

Measurements were made according to the EACVI/ASE recommendations^18^. LA antero-posterior diameter was measured from the parasternal long axis view^19^. LA volume (LAv) was estimate from the LA diameter (LAd) in centimeters, based on an elliptic model, by a best-fitting validated nonlinear equation^20^:

$$LAv=LA{d}^{2.071}\times 2.323$$

LAv was normalized by height in meters to the second power, as recently indicated and prognostically validated in a large cohort study^16^. LAv dilatation was adjudicated for LAV ≥17.5 ml/m^2^ in men or ≥14.8 ml/m^2^ in women, based on the 95th sex-specific percentile of our healthy population participating in the multicenter EchoNoRMAL Study^18,21^. LV mass was estimated from a necropsy-validated formula and normalized for height in meters to the power of 2.7 (LVMi)^22–24^. LV hypertrophy (LVH) was defined as LVMi ≥50 g/m^2.7^ in men and ≥47 g/m^2.7^ in women^22,23^. LV volumes were estimated by the z-derived method^25^, and used to compute ejection fraction and stroke volume^26^.

### Carotid ultrasound

Carotid ultrasound was performed with the patients in the supine position and the neck extended in mild rotation. Examinations were recorded on S-VHS videotapes and analyzed as previously described^1,27^. The maximal arterial intima-media thickness (IMT) was estimated offline in up to 12 arterial walls, including the right and the left, near and far distal common carotid (1 cm), bifurcation, and proximal internal carotid artery, and using an image-processing dedicated workstation (MediMatic, Genova, Italy). Evidence of IMT value higher than 1.5 mm was considered as ‘plaque’^28–33^.

### Statistical analysis

Data were analyzed using SPSS (version 23.0; SPSS, Chicago, IL) and expressed as mean ± 1 SD. Due to the presence of one factor in all participants (hypertension), we considered a CHA_2_DS_2_-VASc ≥3 as representative of high risk sub-population (corresponding to an annual risk of stroke of 3.2%), whereas a score <3 was considered at low-moderate risk. ANOVA was used to compare baseline characteristics of patients with high and low-moderate CHA_2_DS_2_-VASc. The chi-square distribution was used to compare categorical variables, with the MonteCarlo simulation to obtain precise p values. LV mass index, LAVI and IMT were also dichotomized according to the presence of LV hypertrophy, LA dilatation and carotid plaque. Cumulative incidence of AF during follow-up was estimated by product limit Kaplan –Meier survival function, to compare AF-free survival in groups of patients with low-moderate CHA_2_DS_2_-VASc and normal LAVI, high CHA_2_DS_2_-VASc and normal LAVI, low-moderate CHA_2_DS_2_-VASc and dilated LAVI and with coexistence of both high CHA_2_DS_2_-VASc and dilated LAVI.

We calculated hazard ratios (HR) and 95% confidence intervals (CI), by multivariable Cox proportional hazard regression models, using an enter model building procedure with hierarchical steps. The null hypothesis was rejected at a two-tailed α-value of ≤0.05.

## Results

### Patient characteristics

Table 1 shows the CHA_2_DS_2_-VASc score used to classify patients.

**Table 1:** CHA_2_DS_2_-VASc score.

**C**ongestive heart failure/LV dysfunction	1
**H**ypertension	1
**A**ge ≥75 y	2
**D**iabetes mellitus	1
**S**troke/TIA/TE	2
**V**ascular disease (prior MI peripheral artery disease or aortic plaque)	1
**A**ge 65–74 y	1
**S**ex **c**ategory (i.e. female gender)	1

LV = left ventricular; TE = thromboembolism.

At baseline, 956 (15%) patients exhibited high baseline CHA_2_DS_2_-VASc score; they were older, most likely to be women, obese and diabetic, exhibited lower GFR, and higher prevalence of LVH, LAVI dilatation and carotid plaque (p < 0.005) (Table 1). Prevalent Stroke/TIA was found only in the subgroup with high baseline CHA_2_DS_2_-VASc score and not in the group with low-moderate CHA_2_DS_2_-VASc score. During a median follow-up of 54 months, a first episode of AF was identified in 121 patients, significantly more often in those with high CHA_2_DS_2_-VASc (p < 0.0001, Table 2).

**Table 2:** Characteristics of patients with low and high CHA_2_DS_2_-VASc.

Variable	CHA_2_DS_2_-VASc <3 (# 5641)	CHA_2_DS_2_-VASc ≥3 (#956)	p
Age (years)	52 ± 10	67 ± 8	<0.0001
Sex (female %)	37	79	<0.0001
Diabetes (%)	5	43	<0.0001
Obesity (%)	25	32	<0.0001
Stroke/TIA/thromboembolism history (%)	0	6	—
Vascular disease history (%)	0	3	—
Glomerular filtration rate (EPI ml/min/1.73 m^2^)	82 ± 15	69 ± 16	<0.0001
LV Hypertrophy (%)	35	65	<0.0001
LAVI Dilated (%)	10	39	<0.0001
Carotid plaque (%)	43	71	<0.0001
Incident AF (%)	2	4	<0.0001

AF: atrial fibrillation; LAVI: left atrial volume index; LV: left ventricular.

### Incident AF at follow-up

Absolute risk for incident AF was 8.8/1000 patients/year in subjects with high CHA_2_DS_2_-VASc score vs 4.4/1000 patients/year in those with low-moderate CHA_2_DS_2_-VASc score, with an Odds Ratio of 2.57 (95% Cl 1,71–4,86, p < 0.0001).

**Figure 1:**
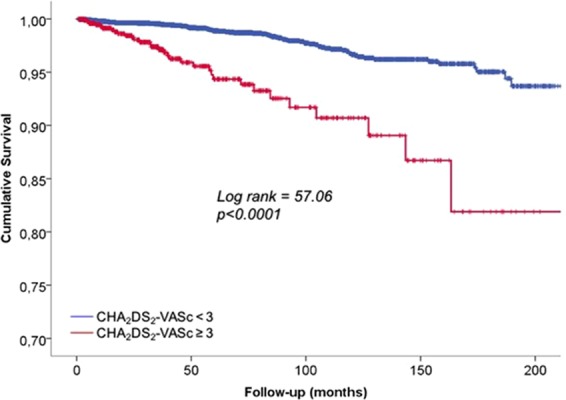
Kaplan-Meier analysis: atrial fibrillation-free survival according to categorized CHA_2_DS_2_-VASc at baseline.

In the Kaplan-Meier plot, AF-free survival was significantly reduced in patients with high CHA_2_DS_2_-VASc score (p < 0.0001, Fig. 1).

The multivariable Cox analysis confirmed that patients with high CHA_2_DS_2_-VASc score had increased hazard of incident AF (HR = 2.99; 95%CI = 1.64–5.43; p < 0.0001), independently of significant effect of LA dilatation (HR = 2.41; 95%CI = 1.35–4.29; p < 0.003). When other markers of target organ damage, including GFR, LVH and carotid plaque, the relative contribution of high CHA_2_DS_2_-VASc score and LA dilatation remained similar and very significant (Table 3).

**Table 3:** Predictors of AF development by Multivariate Cox regression Analysis. Model 1 was adjusted for sex and age; *model 2* included GFR and *model 3* also LVH and carotid plaque.

Variable	Model 1		Model 2		Model 3	
	p	HR (95%Cl)	p	HR (95%Cl)	p	HR (95%Cl)
High CHA_2_-DS_2_-VASC and Normal LAVI	<0.0001	2.99 (1.64–5.43)	0.001	2.77 (1.50–5.12)	0.002	2.63 (1.41–4.89)
Low CHA_2_-DS_2_-VASC and Dilated LAVI	0.003	2.41 (1.35–4.29)	0.004	2.36 (1.33–4.21)	0.012	2.13 (1.18–3.85)
High CHA_2_-DS_2_-VASC and Dilated LAVI	<0.0001	8.54 (5.14–14.19)	<0.0001	7.74 (4.51–13.27)	<0.0001	6.57 (3.70–11.65)
GFR (ml/m/m21.73)			0.291	0.99 (0.98–1.01)	0.384	0.99 (0.98–1.01)
LVH					0.106	1.39 (0.93–2.08)
Carotid Plaque					0.815	1.05 (0.71–1.54)

GFR = glomerular filtration rate; LAVI: left atrial volume index; LVH: left ventricular hypertrophy.

**Figure 2:**
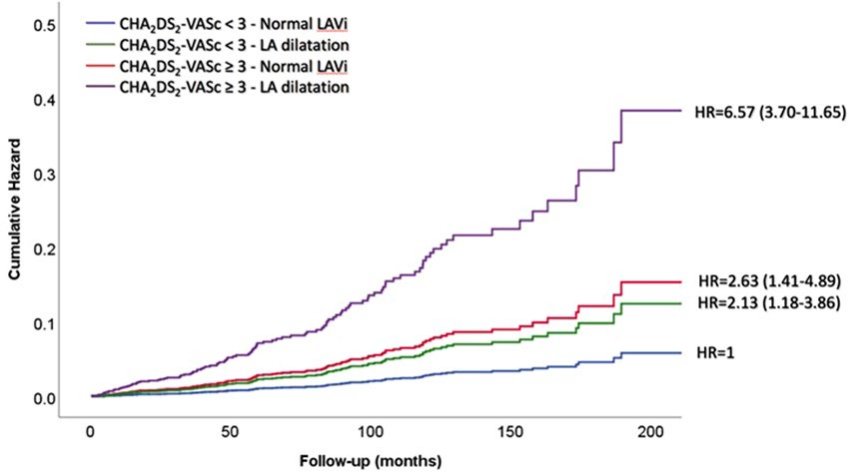
Cumulative hazard of incident AF in different subgroups according to CHA_2_DS_2_-VASc score and left atrial dimension.

Table 3 also shows that there was a progressive increase in the hazard ratio from the subgroup with normal CHA_2_DS_2_-VASc score but dilated left atrium to the subgroup with high CHA_2_DS_2_-VASc score and normal LAD, up to the subgroup carrying both abnormalities, exhibiting more than eight-fold higher hazard than patients with low-moderate CHA_2_DS_2_-VASc score and normal LAD (Table 3 and Fig. 2). This result remained similar also including GFR, LVH and carotid plaque as covariables.

## Discussion

Our study demonstrates that in a large population of hypertensive treated patients:

1. CHA_2_DS_2_-VASc score predicts development of AF, independently of markers of target organ damage, including chronic kidney disease, LVH, carotid plaque and LA dilatation.
2. The coexistence of CHA_2_DS_2_-VASc score ≥3 and of LA dilatation has a synergistic effect, amplifying the risk of incident AF.

In a previous large population-based study, CHADS2 and CHA_2_DS_2_-VASc predicted new-onset AF^34^. However, that cohort included a heterogeneous population of individuals 50 years of age or more. Moreover, no information was available on target organ damage. In contrast, our study focused on a specific population of patients with arterial hypertension including any patient above 18 years of age. We also conducted an extensive evaluation of hypertensive target organ damage (i.e. chronic kidney disease, LVH, carotid plaque and LA dilatation).

Thus, we identify a sub-population of hypertensive patients with high risk of AF, potentially needing more aggressive diagnostic and therapeutic strategies. In addition, we combined the CHA_2_DS_2_-VASc score with a marker of target organ damage, LA dilation^13,19^, that is specific for incident AF, and found that the coexistence of high CHA_2_DS_2_-VASc score and of LA dilatation substantially increases the probability of incident AF. This finding could be of great clinical interest. In fact, this is the first paper demonstrating an interaction between CHA_2_DS_2_-VASc and LA dilation with a hazard ratio that almost triplicates when both conditions coexist.

Although the European Society of Cardiology guidelines recommends screening for AF in patients 65 years or older, by pulse palpation, followed by an electrocardiogram (ECG) in those with irregular pulse frequency, unrecognized AF remains an important cause of morbidity and mortality in high-risk patients, strongly encouraging the research of dedicated strategies for early detection^36,35^. In fact, asymptomatic AF detected by implanted devices, is associated with increased risk of ischemic stroke and systemic embolism^37^. Furthermore. hypertensive patients with pacemaker exhibit silent brain infarcts associated with silent AF^38^. More recently, the REVEAL-AF study demonstrated a substantial proportion of undiagnosed AF (nearly 30%) in patients demographically at high risk of both AF and stroke^39^. In cardiac implantable electronic device population, a very recent paper also demonstrated that “atrial high-rate” episodes increase as CHA_2_DS_2_-VASc score increases^40^. However, in this study only a modest ability to identify AF was demonstrated.

This disappointing result is due to several reasons:

1. Limited time span of observation. In our study a much longer follow-up is available.
2. Only CHA_2_DS_2_-VASc score was tested. It is reasonable to speculate that a multi parametric approach would be more accurate. In our analysis CHA_2_DS_2_-VASc score and LAV dilatation synergistically predict incident AF.
3. Patients included so far were heterogeneous. Our population comprised only patients with arterial hypertension which is the most important risk factor for AF development.

New electronic tools might be of great help in the identification of silent AF, however waiting their implementation and spread, asymptomatic AF remains a critical problem in the prevention of stroke.

Our study offers a new tool, helping identification of a specific risk phenotype for AF, using the same score already validated for identification of high risk of stroke in patients with non-valvular AF. We demonstrate that the risk of incident AF is near doubled in hypertensive patients with CHA_2_DS_2_-VASc ≥3 and, especially important for risk stratification, that this risk is independent of coexisting target organ damage, as LVH, carotid plaque and renal function. There are aspects in our analysis that need to be highlighted.

Firstly, as usual, not all patients with ascertained AF episodes were symptomatic and went to hospital settings. The others were detected incidentally at the time of a doctor visit. Thus, it is reasonable that a number of episodes of AF could not be censored. At least one-third of patients with incident AF do not experience any obvious symptoms or noticeable degradation of quality of life^41,42^. Therefore, the initial manifestation of this ‘silent’ AF could be a thromboembolic event^2^, which can be in turn predicted by CHA_2_DS_2_-VASc score. It is relevant that the same score could also predict cardiac event most associated with ischemic stroke or microstroke, indirectly adding evidence to the close link between AF and stroke. Therefore, one could speculate that a more aggressive diagnostic strategy might be the use of an implantable cardiac monitor, in order to obtain an early diagnosis of AF in those at high risk.

Secondly, our work can be used as a hypothesis generating study. In fact, hypertensive patients with high CHA_2_DS_2_-VASc and LA dilatation might need more aggressive strategy to prevent development of AF or at least its consequence (cerebrovascular ischemic events). The possibility to prescribe oral anticoagulation therapy to prevent both silent brain infarcts and clinically evident strokes might be therefore open. *Ad hoc* studies should be performed at least in the population with coexisting CHA_2_DS_2_-VASc score >3 and LA dilatation, to establish the actual incidence of AF (both symptomatic and sub-clinical) and the subsequent need of anticoagulant therapy.

## Conclusions

We demonstrated, in a population of treated hypertensive patients, that combining information from CHA2DS2-VASc score and LA dimensions predict the incidence of AF. Co-existence of high CHA_2_DS_2_-VASc score with LA dilation substantially increases the probability of incident AF and allows to identify a subpopulation of patients potentially needing more aggressive diagnostic and therapeutic strategies.
